# Regional differences of lung parenchyma attenuation after contrast administration in quantitative dual energy chest CT

**DOI:** 10.1186/s12890-026-04277-0

**Published:** 2026-04-16

**Authors:** Christina Schachner, Quirin D. Strotzer, Lucca Scheuermeyer, Sonja Frank, Florian Raab, Stefanie Meiler, Maximilian V. Malfertheiner, Simone Hammer, Christian Stroszczynski, Okka W. Hamer

**Affiliations:** 1 Department of Radiology, University of Regensburg Medical Center, Franz- Josef-Strauss-Allee 11, Regensburg, 93053 Germany; 2 Clinic of Pneumonology, Donaustauf Hospital, Ludwigstrasse 68, Donaustauf, 93093 Germany; 3 Department of Radiology, Donaustauf Hospital, Ludwigstrasse 68, Donaustauf, 93093 Germany

**Keywords:** Dual-energy CT, Lung, Density, Contrast enhancement

## Abstract

**Purpose:**

The administration of contrast material increases the density of lunge parenchyma on computed tomography. Lung pathologies which are characterized by an only slight alteration of lung attenuation (e.g. ground glass opacities or emphysema) should therefore be evaluated on non-enhanced scans in order to avoid misinterpretation. However, contrast administration is very helpful in some clinical scenarios like suspected pulmonary embolism or malignancy. This study aimed to quantify the amount of increase of lung parenchymal density after intravenous contrast administration compared to non-enhanced images and whether this increase exhibits regional variation similar to or different from the attenuation on non-enhanced scans.

**Materials and methods:**

This retrospective, IRB-approved, bi-center study included patients who underwent both contrast-enhanced dual-energy and non-enhanced chest CT scans within a year between 04/2018 and 12/2022. Scans were co-registered and semi-manually segmented into the whole lung and isovolumetric segmentations of the ventral/dorsal halves and upper/middle/lower thirds. Mean lung density for each region was calculated from contrast-enhanced (CE), virtual non-contrast (VNC), and true non-contrast (TNC) scans. Bland-Altman analyses with non-parametric limits of agreement assessed the mean difference in attenuation values, with differences between lung regions tested by the Mann-Whitney U test. Subgroup differences were also analyzed by Mann-Whitney U tests. Correlation analyses were performed to investigate the correlation between the increase in density from contrast media and the non-enhanced density.

**Results:**

Fourty eight patients (26 females, median age: 63y) fulfilled the inclusion criteria. The majority suffered from emphysema and/or overinflation. The mean increase in lung density between TNC and CE scans was 11.65 HU [95% Confidence Interval: 6.10, 17.19] for the whole lung. Amount of increase ranged from 7.79 HU [0.65, 14.94] to 16.35 HU [11.22, 21.53] for different lung regions. Comparing VNC with CE scans showed an average increase in lung density of 11.21 HU [8.63, 13.79]. Amount of increase ranged from 2.02 HU [-2.06, 6.10] to 16.58 HU [13.47, 19.70] for different lung regions. A significant difference between contrast enhancement based on TNC versus VNC images was found for the upper third only. For the whole lung and all other lung regions no differences in density increase were seen. Regression analyses and Spearman’s Rho showed that increase in density tended to be more pronounced in lung regions with higher baseline density in non-enhanced scans.

**Conclusions:**

The mean increase in density of lung parenchyma after contrast administration was approx. 11 HU, for both TNC and VNC baseline images. There were substantial variations across individuals and lung regions with a tendency of higher increases in areas with higher baseline density on non-enhanced images. Knowledge of these phenomena avoid misinterpretation of contrast enhanced scans.

## Key points

Regional quantitative attenuation measurements of contrast-enhanced multi-energy as well as virtual and true non-contrast chest CT scans were compared across 48 patients.

The mean increase of lung parenchyma attenuation after contrast administration was 12 HU (True Non-Contrast; TNC) and 11 HU (Virtual Non-Contrast; VNC). There were substantial differences between individuals.

Baseline non-enhanced density and density increase after contrast administration varied considerably between different lung regions in our patients, the majority of which suffered from emphysema.

The increase in density measured from baseline TNC images was comparable to that measured from VNC images.

## Introduction

Computed tomography (CT) of the lung can reveal numerous pathological patterns. These can be broadly categorized in alteration of the attenuation upwards or downwards relative to healthy lung parenchyma. Among others, increased attenuation is seen in ground glass opacities and consolidation and decreased attenuation in air trapping and emphysema. To detect these pathologies, radiologists rely on their subjective visual impression of the images, also paying attention to altered contrast patterns such as the *black bronchus sign*. Beyond subjective interpretation, quantitative CT has become established e.g. for determining the extent and distribution of pulmonary emphysema.

To accurately assess subtle differences in density—either by observation or measurement—a true non-contrast CT (TNC) is necessary, as contrast material raises lung parenchymal density. This increase can result in incorrect interpretations or imprecise quantitative assessments. However, certain clinical situations—such as suspected pulmonary embolism or malignancy—necessitate the administration of contrast material. Previous research investigating the impact of contrast agent on Hounsfield units (HU) of lung parenchyma has often been limited by small sample size and/or the omission of regional variation [1, 2]. However, even in healthy persons perfusion of the lung is heterogeneous not only but above all influenced by gravity [3]. On top of that lung parenchyma pathology like pulmonary emphysema or interstitial lung disease alters lung perfusion. Thus, it is conceivable that the density of the lung parenchyma after contrast administration does not increase uniformly but instead shows regional differences.

The aim of this study is to determine (1) regional differences of lung density in TNC and virtual non contrast (VNC) images calculated from a dual energy dataset (2), the amount of increase of lung parenchymal density after intravenous contrast administration compared to both TNC and VNC images and (3) whether this increase exhibits regional variation similar to or different from non-enhanced scans.

## Material and methods

### Patients, image acquisition, and analysis

We present a secondary analysis of a study focusing on patients who underwent both multi-energy chest CT and non-contrast chest CT from April 2018 to December 2022 at a university hospital and a tertiary pulmonary medicine facility. The previous study provided a detailed description of patients and dataset preparation. It did not include measurements from the contrast-enhanced scans [Reference removed for anonymization].

In short, contrast-enhanced chest CTs were acquired at either a dual-source Somatom Definition Flash or a split-beam filter Somatom Definition AS+ scanner (both Siemens Healthineers, Erlangen, Germany) in a supine position during end-inspiratory hold. Iohexol (Accupaque 350; GE Healthcare Buchler, Braunschweig, Germany) was intravenously administered at a flow rate of 3–5 ml/s and a dose of 1.5 ml/kg body weight. The scan was triggered using a bolus-tracking technique with a delay of 5s after a region of interest in the main pulmonary artery reached the threshold of 100 Hounsfield Units (HU). All CECT scans were performed as CT pulmonary angiogram. Virtual non-enhanced images were reconstructed using Python (v3.8.12; Python Software Foundation, Wilmington, USA). Non-enhanced chest CTs were acquired at various scanners from Siemens and GE HealthCare (Chicago, Illinois, USA) in supine position and end-inspiratory hold.

Exclusion criteria were lacking complete lung coverage, presence of motion artifacts, acute pathologies (like effusion, pneumonia, congestion), differences in chronic pathologies (like fibrosis, emphysema) between enhanced and non-enhanced scans and a time difference of more than 1 year between the scans.

Images were axially reconstructed at 1 mm section thickness, exported in DICOM format, converted to NIFTI and resampled to a 5 mm resolution in z-direction to facilitate manual control of the segmentations. CECTs and non-contrast scans underwent non-linear co-registration with visual validation by a trained third year medical student (XX) [4]. Lung regions (left, right, upper third, middle third, lower third, ventral half, dorsal half; for further information see [Reference removed for anonymization]) were segmented automatically binarily with a pre-trained neural network. Segmentation results were manually inspected and corrected where needed by XX and finally verified by a radiology resident with four years of experience (YY) [1]. The anatomical segmentations were used to mask CT scans for region-specific analysis, with mean lung density calculated according to established guidelines [2].

A fellowship trained thoracic radiologist (ZZ) with 20 years of experience evaluated all CT scans for pathologic findings. XX and ZZ evaluated all CECT scans to identify streak artifacts produced by contrast material in the superior vena cava or subclavian vein that could potentially influence the HU measurements. The severity of the streak artifacts was evaluated using a four-point scale: 0: no streak artifacts, 1: minimal streak artifacts with no impact on density measurement, 2: streak artifacts with minor focal influence on density measurement, 3: pronounced streak artifacts with major influence on density measurement.

### Statistical analysis

Continuous data are presented as mean with standard deviations (SD) or median with interquartile range, while categorical data are reported as absolute counts and percentages. The normality of the data distribution was assessed using histograms and Shapiro-Wilk tests, and the equality of variances was evaluated with Levene’s test.

MLDs were compared through Bland-Altman analysis. Mean differences were determined as CECT minus TNC (HU_CECT_ – HU_TNC_) and VNC (HU_CECT_ – HU_VNC_), respectively.

Mann-Whitney-U tests were employed to compare the MLDs of the subgroups in VNC and TNC scans, and to evaluate the subgroup differences of CECT-VNC and CECT-TNC.

To investigate the correlation between the increase in density from contrast media and the non-enhanced density, regression plots were generated and Spearman’s Rho was determined.

Given that the pairwise differences did not conform to a normal distribution, non-parametric limits of agreement for the Bland-Altman analysis were established as the 2.5th and 97.5th percentiles [5].

Data analysis and visualization were performed using IBM SPSS Statistics version 26.

## Results

### Patient characteristics

Forty-eight patients met the inclusion criteria (26 females and 22 males, median age: 63 years [interquartile range: 58–69]). Most patients suffered from emphysema and/or overinflation (30/48). One patient had a fibrotic interstitial lung disease. 10/48 patients showed postinfectious residua. Lung parenchyma was normal in 7/48 patients.

### Streak artifacts

36 scans showed no streak artifacts (scale 0), 8 scans showed minimal artifacts (scale 1), 4 scans showed streak artifacts with minor focal influence on density measurement (scale 2), and no scan showed pronounced artifacts (scale 3).

### Differences in lung attenuation

**Fig. 1:**
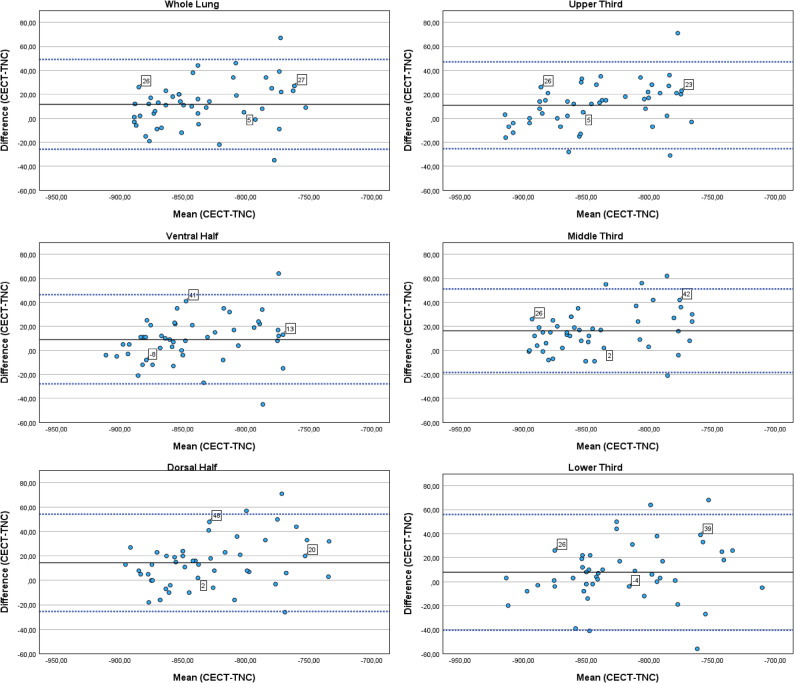
Density increase of lung parenchyma after contrast administration with baseline density measured on TNC images (CECT-TNC). Blue dotted horizontal lines represent the limits of agreement, black horizontal lines represent the mean difference. Numbers in squares show example HU values of the differences (CECT-TNC). Bland–Altman analyses

**Fig. 2:**
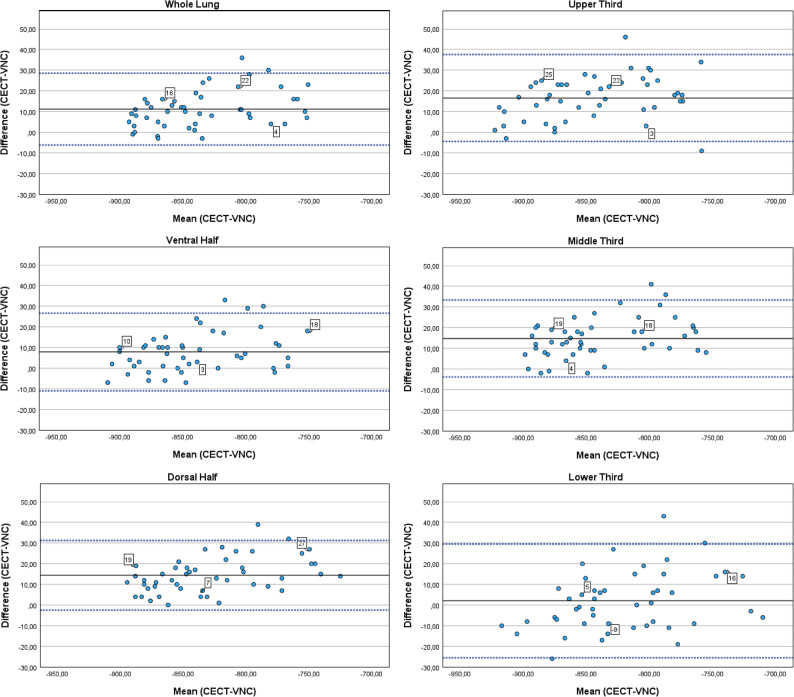
Density increase of lung parenchyma after contrast administration with baseline density measured on VNC images (CECT-VNC). Blue dotted horizontal lines represent the limits of agreement, black horizontal lines represent the mean difference. Numbers in squares show example HU values of the differences (CECT-VNC). Bland–Altman analyses

Table 1 summarizes the average mean lung density for the CECT, VNC, and TNC images. Results of the Bland-Altman analyses are reported in Table 2, and Figs. 1 and 2.

**Table 1:** Mean ± standard deviation of quantitative CT measurements for contrast-enhanced (CECT), virtual non-contrast (VNC), and true non-contrast (TNC) scans

	Mean CECT MLD [HU]	Mean VNC MLD [HU]	Mean TNC MLD [HU]
Whole Lung	-829 ± 46	-840 ± 42	-840 ± 40
Upper Third	-836 ± 50	-853 ± 46	-847 ± 42
Middle Third	-832 ± 46	-847 ± 42	-848 ± 40
Lower Third	-817 ± 53	-819 ± 48	-825 ± 47
Ventral Half	-837 ± 45	-845 ± 41	-846 ± 40
Dorsal Half	-821 ± 49	-835 ± 45	-835 ± 42

Units in square brackets

*Abbreviations*: *MLD* Mean lung density

Concerning the entire lung, a mean difference of 11.65 HU [6.10, 17.19] was identified in MLD between TNC and CECT scans (Table 2). When examining different lung regions, these mean differences varied with values ranging from 7.79 [0.65, 14.94] HU to 16.35 [11.22, 21.53] HU.

**Table 2:** Bland–Altman analyses (mean differences with 95% confidence intervals and limits of agreement) of density in contrast-enhanced (CECT) minus density in true non-contrast (TNC) images and virtual non-contrast (VNC) images, respectively

	Mean Difference CECT-TNC [95% CI]	Limits of Agreement (CECT-TNC)	Mean Difference CECT-VNC [95% CI]	Limits of Agreement (CECT-VNC)	*P* value
Whole Lung	11.65 [6.10, 17.19]	-25.81, 49.10	11.21 [8.63, 13.79]	-6.17, 28.58	0.86
Upper Third	10.90 [5.51, 16.24]	-25.34, 47.13	16.58 [13.47, 19.70]	-4.45, 37.62	0.04
Middle Third	16.35 [11.22, 21.53]	-18.42, 51.13	14.75 [11.99, 17.51]	-3.86, 33.36	0.48
Lower Third	7.79 [0.65, 14.94]	-40.44, 56.02	2.02 [-2.06, 6.10]	-25,52, 29.56	0.07
Ventral Half	9.00 [3.52, 14.48]	-27.96, 45.96	7.90 [5.10, 10.69]	-10.97, 26.76	0.66
Dorsal Half	14.44 [8.54, 20.34]	-25.40, 54.27	14.44 [11.94, 16.93]	-2.40, 31.28	> 0.99

Similarly, for VNC compared to CECT scans, the overall lung showed a mean difference of 11.21 [8.63, 13.79] HU (Table 2). Within the subgroups, these differences spanned from 2.02 [-2.06, 6.10] HU to 16.58 [13.47, 19.70] HU.

A significant difference between contrast enhancement based on TNC versus VNC images was found for the upper third only (Table 2). For the whole lung and all other lung regions no differences in density increase were seen.

**Fig. 3:**
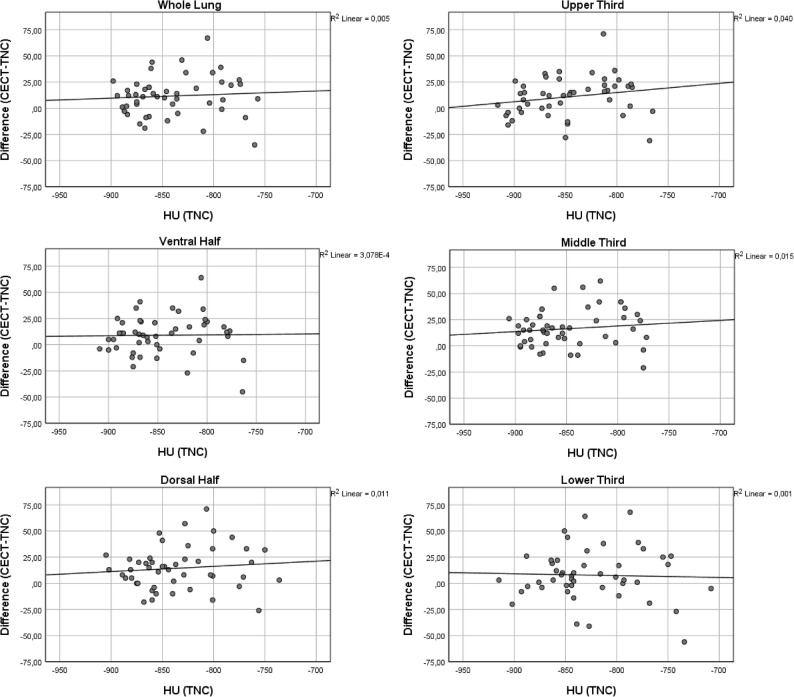
Correlation of baseline density on true non-contrast (TNC) images with density increase (CECT-TNC) after contrast administration. CECT: contrast enhanced CT

**Fig. 4:**
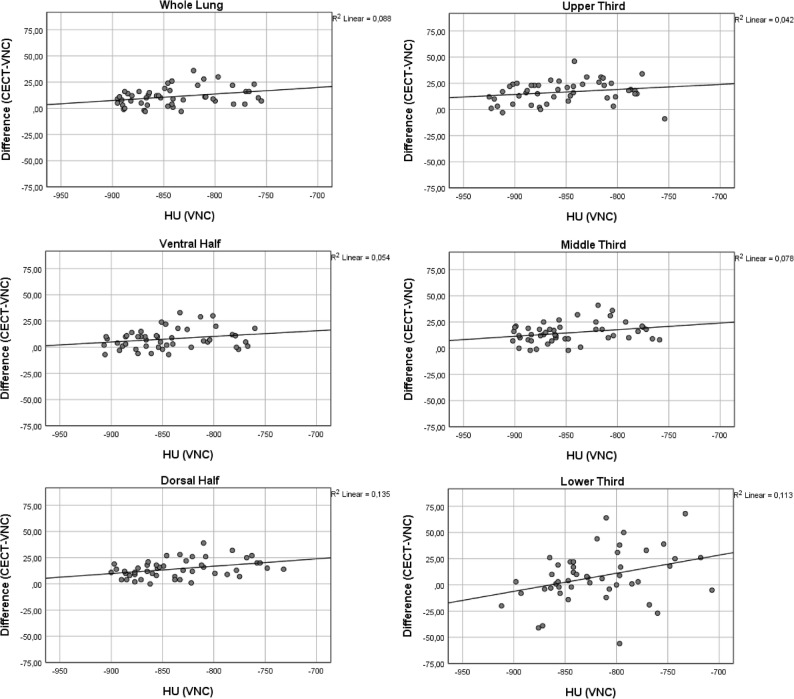
Correlation of baseline density on virtual non-contrast (VNC) images with density increase (CECT-VNC) after contrast administration. CECT: contrast enhanced CT

**Fig. 5:**
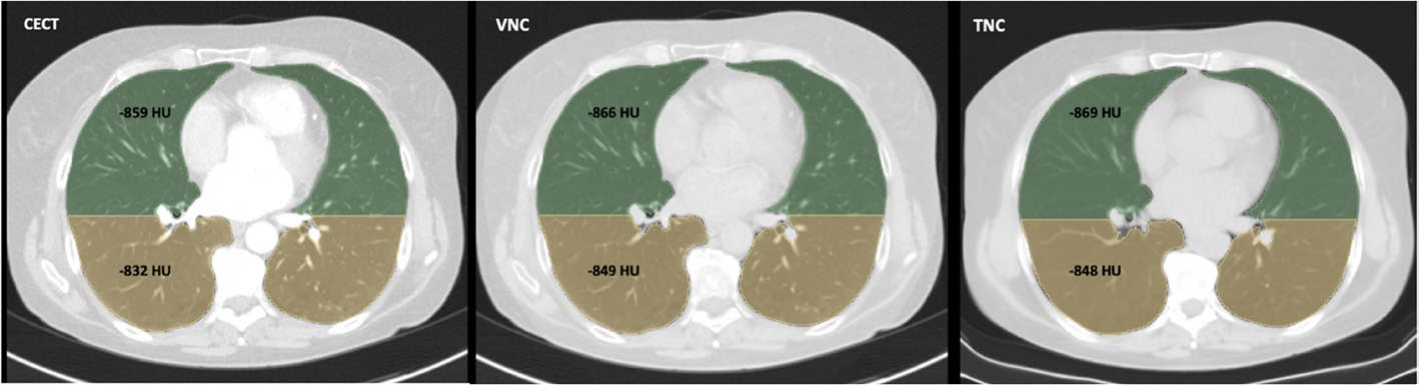
Axial CT scans of an exemplary patient: contrast-enhanced (left), virtual non-contrast (VNC) (middle), true non-contrast (TNC) (right); colored visualization of ventral and dorsal isovolumetric segmentation of the lung with annotations of the mean lung density (MLD) in HU. The example shows a greater increase in density at higher baseline HU and only minimal differences between VNC and TNC images

The density in the non-enhanced scans (both TNC and VNC) differed significantly between the upper and lower as well as the middle and lower third (Table 3). Also, the extent of density increase varied significantly between the upper and middle third, middle and lower third and ventral and dorsal half for VNC images (Table 4). For TNC images the density increase differed significantly between the middle and lower third only (Table 4). Correlation analyses demonstrated that increase in density was higher in lung regions with higher baseline density in non-enhanced scans (Figs. 3 and 4). This finding was more pronounced for VNC than TNC images. However, statistical analysis demonstrated a light to middle correlation only (Spearman’s Rho 0.22–0.39 for VNC; 0.02–0.26 for TNC; Table 5) (Fig. 5).

**Table 3:** Differences of lung density between different lung regions for virtual non-contrast (VNC) and true non-contrast (TNC) scans

	*P* Value (VNC)	*P* Value (TNC)
Upper Third - Middle Third	0.50	0.85
Upper Third - Lower Third	0.001	0.03
Middle Third - Lower Third	0.003	0.01
Ventral Half - Dorsal Half	0.32	0.15

**Table 4:** Differences of density increase between different lung regions after contrast administration for baseline virtual non-contrast (VNC) and true non-contrast (TNC) images

	*P* Value (CECT-VNC)	*P* Value (CECT-TNC)
Upper Third - Middle Third	0.24	0.22
Upper Third - Lower Third	< 0.001	0.41
Middle Third - Lower Third	< 0.001	0.046
Ventral Half - Dorsal Half	< 0.001	0.27

*CECT* Contrast enhanced CT

**Table 5:** Spearman’s Rho for HU (VNC) to difference (CECT-VNC) and HU (TNC) to difference (CECT-TNC)

	*r*_s_ (VNC)	*r*_s_ (TNC)
Whole Lung	0.28	0.14
Upper Third	0.26	0.26
Middle Third	0.25	0.13
Lower Third	0.22	0.02
Ventral Half	0.23	0.14
Dorsal Half	0.39	0.14

The differences between the VNC and TNC images have been published elsewhere [Reference removed for anonymization].

## Discussion

Non-enhanced CT is preferable for detecting certain pulmonary pathologies like ground glass opacities and pulmonary emphysema since intravenous contrast administration increases lung parenchymal density. Non-contrast CT is even mandatory for quantitative CT analysis. Some clinical indications, however, - such as suspected pulmonary embolism - require contrast administration. Misinterpretations in terms of missed pathology or false positive readings might occur when the effect of contrast administration on lung parenchyma attenuation is neglected. The present study compares contrast-enhanced CT (CECT) with true (TNC) and virtual (VNC) non-enhanced images intraindividually. In contrast to previous studies, we performed coregistration of images and evaluated 5 different lung regions to capture potential regional differences in non-contrast attenuation as well as regarding the amount of density increase after contrast enhancement. The CTs of the patient cohort comprised a magnitude of pathologies with pulmonary emphysema being the most frequent one.

TNC and likewise VNC images demonstrated profound regional density variations within the lung. This heterogeneity is well explained by the composition of the patient cohort the majority of which presented with pathological lung findings, especially pulmonary emphysema.

The density increase after contrast administration was mean 11 and 12 HU (based on VNC and TNC, respectively). The wide confidence intervals and limits of agreement indicate substantial differences between individuals. Moreover, the increase in density after contrast administration was different between lung regions, both for TNC but especially VNC images. Hereby, the amount of density increase tended to be higher in lung regions with higher density on non-enhanced images. We hypothesize that this observation might be attributable to the impact of hypoxic vasoconstriction in poorly aerated lung regions within our cohort, which predominantly consisted of individuals with emphysema or airway disease. Blood flow and consecutively contrast media is directed into relatively well aerated lung parenchyma and away from lung regions with impaired gas exchange to avoid functional shunting. Radiologists have to be aware of this dynamic to avoid false positive reports of ground glass opacities in CECTs of patients with emphysema or obstructive airways disease. Also, quantitative analyses of lung pathology, e.g., in interstitial lung disease, can be distorted in contrast-enhanced scans and should therefore be performed without contrast whenever possible.

There is limited research comparing CECT and non-contrast CT in quantitative chest imaging. Thoo et al. found that lung parenchyma density increased by an average of 24 HU in 28 patients using dual-energy post-contrast scans and VNC data. However, their findings are constrained by the small sample size, absence of TNC scans, HU measurement within manually placed small FOVs rather than lung segmentation and the lack of control for variables such as different contrast phases [6]. In our study, the entire lung was segmented and all CECTs were triggered in the pulmonary trunk to ensure consistency of timing. This approach is essential as variations in contrast timing affect lung parenchyma attenuation [7]. Moreover, several studies have demonstrated minor inaccuracies in estimating iodine-associated attenuation using VNC algorithms, despite a generally high correlation [8].

Heussel et al. studied 12 patients to assess whether CECT is comparable to non-enhanced scans for emphysema quantification. They found that contrast application resulted in an average increase of 18 HU in median lung density which is considerably more than in our study [9]. The difference could be based on differences in the study setup. Our cohort consisted predominantly of patients with emphysema/overinflation. Heussel et al. do not specify lung pathology, but note that five out of 12 patients had an ILD. Lung pathology clearly influences enhancement characteristics. Moreover, Heussel et al. used a fixed 100 ml dose of contrast material with 300 mg iodine per milliliter, whereas our cohort received a body weight–adjusted dose containing 350 mg iodine per milliliter.

Our study has limitations. The significant time interval between CT scans may have affected results due to disease progression or acute pathologies. However, acquisitions were meticulously reviewed, excluding scan pairs with even minor pathology differences. Nonetheless, we cannot entirely exclude the possibility that minor changes may have remained undetected. This potential source of error would have only influenced the comparison between the TNC and CECT/VNC images. However, our analysis revealed that the VNC and TNC values, as well as the density increases, were nearly identical, suggesting no significant pathological changes between the CT scans.

As for lung regions, segmentation of pulmonary lobes would seemingly have been ideal. We intentionally avoided this approach due to anatomical challenges, opting for lung thirds, a well-established and reliable standard.

The kilovolt peak (kVp) setting influences HU measurements. However, only the TNC data might have been affected by this variation, as the VNC and CECT data were derived from the weighted average images of the same scan. As for the TNC images, it should be noted that the effect of kVp on HU is tissue-dependent and is particularly pronounced in the presence of contrast media and in solid tissues. For lung parenchyma, however, this effect is negligible.

The study is also constrained by its relatively small sample size. Yet, a key aspect of our work was to highlight the variability in lung parenchyma enhancement. This finding remains valid regardless of the study group size.

In summary, this study found that regional lung density varies significantly due to underlying pulmonary pathology. The increase in density following contrast administration also showed substantial variation across individuals and lung regions. Density increase tended to be higher in areas with greater baseline density on non-enhanced images. This phenomenon might be attributable to mosaic perfusion resulting from hypoxic vasoconstriction, as most participants in our cohort had pulmonary emphysema. Radiologists should understand how pulmonary pathology affects lung density and recognize that pathology can lead to uneven contrast distribution and consecutive possible misinterpretation.

## Data Availability

The datasets used and analyzed during the current study are available from the corresponding author on reasonable request.

## Abbreviations

CECT: Contrast-Enhanced CT
HU: Hounsfield Units
MLD: Mean Lung Density
TNC: True Non-Contrast
SD: Standard Deviation
VNC: Virtual Non-Contrast
